# Phylogeny- and morphology-based recognition of new species in the spider-parasitic genus *Gibellula* (Hypocreales, Cordycipitaceae) from Thailand

**DOI:** 10.3897/mycokeys.72.55088

**Published:** 2020-09-02

**Authors:** Wilawan Kuephadungphan, Kanoksri Tasanathai, Booppa Petcharad, Artit Khonsanit, Marc Stadler, J. Jennifer Luangsa-ard

**Affiliations:** 1 National Center for Genetic Engineering and Biotechnology (BIOTEC), 113 Thailand Science Park, Phahonyothin Road, Khlong Nueng, Khlong Luang, Pathum Thani 12120 Thailand National Center for Genetic Engineering and Biotechnology (BIOTEC) Pathum Thani Thailand; 2 Department of Biotechnology, Faculty of Science and Technology, Thammasat University, Pathumthani 12120 Thailand Thammasat University Pathumthani Thailand; 3 Department of Microbial Drugs, Helmholtz Centre for Infection Research, 38124 Braunschweig, Germany Helmholtz Centre for Infection Research Braunschweig Germany

**Keywords:** Cordycipitaceae, *
Gibellula
*, spider specialist fungus, taxonomy

## Abstract

Thailand is known to be a part of what is called the Indo-Burma biodiversity hotspot, hosting a vast array of organisms across its diverse ecosystems. This is reflected by the increasing number of new species described over time, especially fungi. However, a very few fungal species from the specialized spider-parasitic genus *Gibellula* have ever been reported from this region. A survey of invertebrate-pathogenic fungi in Thailand over several decades has led to the discovery of a number of fungal specimens with affinities to this genus. Integration of morphological traits into multi-locus phylogenetic analysis uncovered four new species: *G. cebrennini*, *G. fusiformispora*, *G. pigmentosinum*, and *G. scorpioides*. All these appear to be exclusively linked with torrubiella-like sexual morphs with the presence of granulomanus-like asexual morph in *G. pigmentosinum* and *G. cebrennini*. A remarkably high host specificity of these new species towards their spider hosts was revealed, and for the first time, evidence is presented for manipulation of host behavior in *G. scorpioides*.

## Introduction

To arthropodologists or even arachnologists, it is surprising that fungal pathogens of spiders seem to be generally neglected when the host can be completely overgrown by the pathogens to be unrecognizable as a spider. Nonetheless, this group of fungi has been known and studied for more than two centuries (Evans 2013). Recently, over 80 fungal species, mostly distributed in Cordycipitaceae, have been reported as spider pathogens (Shrestha et al. 2019). Among them, only *Gibellula* and *Hevansia* are obligate parasites of spiders whereas others appear to be natural enemies of different insects and do not show apparent host specificity.

*Gibellula* is well-known to be a specialized spider-parasitic genus widely distributed worldwide, mostly found in tropical regions (Shrestha et al. 2019). Originally, the type species, *G pulchra* (Sacc.) Cavara was known as *Corethropsis pulchra* Sacc. collected from Italy, recognized by producing primarily synnematous, aspergillus-like conidiophores with terminal vesicles, each gives rise to phialides produced on metulae (Saccardo 1877; Shrestha et al. 2019). After establishing a genus *Gibellula* Cavara in honor of Prof. Giuseppe Gibelli by Cavara (1894), a number of species in this genus were recorded across the world (Shrestha et al. 2019). Currently, nearly 40 species have been described and listed in the global fungal databases Index Fungorum (www.indexfungorum.org) and MycoBank (www.mycobank.org). According to the review of Shrestha et al. (2019), many of them including *G arachnophila* (Ditmar) Vuill., G arachnophila f. arachnophila (Ditmar) Vuill., G arachnophila f. macropus Vuill., *G. aranearum* P. Syd., *G globosa* Kobayasi & Shimizu, *G globosostipitata* Kobayasi & Shimizu, *G haygarthii* Van der Byl, *G suffulta* Speare and *G tropicalis* Sawada were synonymized with *G pulchra* whereas G arachnophila f. leiopus Vuill. ex. Maubl., *G araneae* Sawada and *G perexigua* (Kobayasi) Koval were synonymized with *G leiopus* (Vuill. ex Maubl.) Mains. In addition to these species, the identities of several other species reported in this genus still remain doubtful. Petch (1932) expressed uncertainty about the identities of *G aspergilliformis* (Rostr.) Vuill. and *G phialobosia* Penz. & Sacc. by pointing out that the narrow metulae and spherical conidia in chains present in *G aspergilliformis* and the flask-shaped phialides in the latter species were not common features of *Gibellula*. Moreover, description of *G eximia* Höhn. did not point to the genus. Since *Gibellula* is well-known as an obligate parasite of spiders, Mains (1950) reported that the assignment of *G elegans* Henn. to this genus might be erroneous, as this species is found occurring on locusts. According to Mains (1950), the description of *G capillaris* Morgan did not fit the concept of *Gibellula* and re-examination of the type specimen is unfortunately infeasible since it is no longer in a good condition. Tzean et al. (1997) doubted the identity of *G araneicola* Sawada that produces an isarioid morph instead of *Gibellula*. In the case of *G. petchii* Humber & Rombach, it is still unclear whether the species name should be retained or abandoned. *Gibellula petchii* Humber & Rombach was proposed to accommodate *Cylindrophora aranearum* Petch, which was originally described as the conidial state of *Torrubiella albolanata* Petch and later elevated to generic rank as a new genus, *Granulomanus* de Hoog & Samson (de Hoog 1978; Humber and Rombach 1987; Petch 1944). From the point of view of Humber and Rombach (1987), *Granulomanus* should be synonymized with *Gibellula* as it almost never occurs in the absence of *Gibellula* and/or its torrubiella-like sexual morph. *Cylindrophora aranearum* (≡*Granulomanus aranearum* (Petch) de Hoog & Samson) was henceforth synonymized with *G. petchii*. On the other hand, Samson and Evans (1992) argued that *Granulomanus* naturally occurs independently on spider hosts either with or without *Gibellula*. Thus, the genus should be retained as an independent asexually typified genus resulting in rejection of *G. petchii.* According to a recent taxonomic revision of the Cordycipitaceae, which was largely based on molecular data, several generic names including *Granulomanus* were suppressed (Kepler et al. 2017). Nevertheless, the taxonomic dilemma of *G. petchii* cannot yet be resolved owing to the lack of its sequence data. Based on these facts, only 17 species have been accepted in *Gibellula* (Shrestha et al. 2019).

Thailand is one of the most biodiverse countries in Southeast Asia and the BIOTEC culture collection has more than 700 *Gibellula* strains. Despite this number, only very few *Gibellula* species with distinct features could be recognized morphologically (Luangsa-ard et al. 2008, 2010). *Gibellula gamsii* is the most recently described species reported from Thailand (Kuephadungphan et al. 2019).

Our continuous survey of invertebrate-pathogenic fungi in Thailand for over two decades has led to the BIOTEC Bangkok Herbarium (BBH) and the BIOTEC Culture Collection (BCC) owning a very large herbaria, and culture collections, which greatly facilitates the exploration of existing species including *Gibellula*. Here, phylogeny within *Gibellula* species from the ribosomal internal transcribed spacer (ITS) regions analyzed prior to this study enabled recognition of four distinct clades. The morphological and multi-gene phylogenetic data confirm their identities as well as taxonomic placements. Herein, new species are described that are illustrated morphologically and phylogenetically and compared with other species in the same genus.

## Materials and methods

### Collection of fungal materials and isolation of pure cultures

Spiders parasitized by *Gibellula* spp. firmly attached on the underside of living leaves were collected from various locations throughout Thailand, mostly in the Northeastern region. The leaf bearing the parasitized spider was carefully detached from the tree, placed in a plastic box and transported to the laboratory for immediate isolation of a pure culture according to the protocols described by Kuephadungphan et al. (2014) and Mongkolsamrit et al. (2018). Briefly, the conidia located on the synnemata were gently swiped with small agar plugs of potato dextrose agar (PDA) which were then placed on a PDA plate. The conidia were allowed to germinate at 25 °C for a few days. Thereafter, each agar plug with actively growing mycelia was transferred to a fresh PDA plate where the fungus could readily grow for another 6–8 weeks. For specimens bearing sexual morphs, pure cultures were isolated by enabling ascospores from the perithecia to be discharged onto PDA plates and allowing them to grow at 25 °C for a certain amount of time depending on the growth rate of each individual strain. All cultures were required to be deposited in the BCC, Thailand while the fungal specimens were dried in an electric food dryer (50–55 °C) before being stored at the BBH, Thailand.

### Morphological characterization

Microscopic characteristics were studied based on observation of synnemata and perithecia. Each of them was detached from the stroma and mounted on a microscope slide containing a drop of lactophenol cotton blue solution. Shapes and sizes of individual character were determined and measured according to Mongkolsamrit et al. (2018).

### Identification of spider hosts

The mummified spiders were identified based on morphological characteristics. To better understand the host-pathogen relationship, posture of spider at attachment on leaf surface (touching or lifting), position of spider on the leaf (under or upper side), and leaf type (monocots or dicots) were herein recorded.

### Molecular phylogenetic analyses

DNA extraction, PCR amplification of five DNA regions as well as purification of PCR products were conducted according to the protocols previously described by Kuephadungphan et al. (2019). The PCR amplicons were obtained using primers ITS1F (Gardes and Bruns 1993) and ITS4 (White et al. 1990) for nuc rDNA ITS1-5.8S-ITS2 (ITS barcode), LR5 or LR7 (Vilgalys and Hester 1990) and LROR (Bunyard et al. 1994) for the partial region of nuc 28SrDNA (LSU), 983F and 2218R (Rehner and Buckley 2005) for translation elongation factor 1-alpha (*TEF1*), RPB1-Ac and RPB1-Cr (Murata et al. 2014) for the largest subunit of RNA polymerase II (*RPB1*) and fRPB2-5F and fRPB2-7cR (Liu et al. 1999) for the second largest subunit of RNA polymerase II (*RPB2*).

DNA sequences were checked manually for ambiguous base calls and all sequences were assembled using BioEdit v.7.2.5 (Hall 1999; Hall et al. 2011). Sequence alignment was conducted using MAFFT 7.017 with G-INS as the algorithm and default settings used for gap opening and gap extension penalties (Katoh and Toh 2008). Manual adjustments were subsequently made in BioEdit. Concatenation of multiple loci was performed in GENEIOUS® 7.1.19 (http://www.geneious.com, Kearse et al. 2012).

Phylogenetic relationships were inferred using maximum likelihood (ML) with GTRCAT as the substitution model in RAXML 7.2.8 (Stamatakis 2006) and the rapid bootstrap analysis algorithm. Relative support for the branches was obtained from bootstrap analysis with 1,000 replicates. Bayesian analysis was performed according to Mongkolsamrit et al. (2019) using MRBAYES v.3.2.7 (Ronquist and Huelsenbeck 2003) on XSEDE via the online CIPRES Science gateway using SYM+G selected by MRMODELTEST 2.2 (Nylander 2004) as the best nucleotide substitution model. Posterior probabilities were performed by Markov Chain Monte Carlo Sampling (MCMC) in which four chains were run for 5,000,000 generations with a tree sampling frequency of 100 and a burn-in of 10% of the total run.

## Results

### Molecular phylogeny

**Figure 1.**
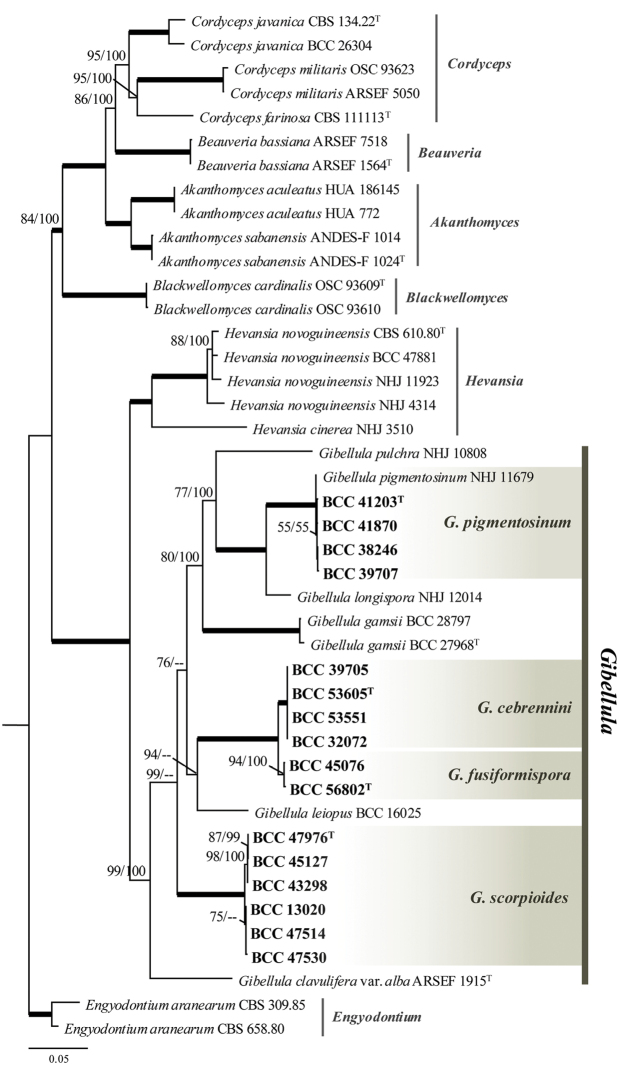
Phylogenetic tree inferred from a RAxML search of a concatenated alignment of ITS, LSU, *TEF1*, *RPB1* and *RPB2* showing the relationship among *Gibellula* and related genera. Bootstrap proportions/ Bayesian posterior probabilities ≥ 50% are provided above corresponding nodes; nodes with 100% support are shown as thick lines. The ex-type strains are marked with a superscript T (^T^) and the isolates reported in this study are bold.

The combined data set of 43 taxa (Table 1) comprised 4,325 characters including 680, 859, 917, 1,109 and 917 characters derived from ITS, LSU, *TEF1*, *RPB1* and *RPB2*, respectively with *Engyodontium aranearum* as the outgroup. The multigene tree (Fig. 1) comprised seven different genera belonging to the family Cordycipitaceae including *Akanthomyces*, *Beauveria*, *Blackwellomyces*, *Cordyceps*, *Engyodontium*, *Gibellula* and *Hevansia*. The analyses showed the genera segregated corresponding to the recent phylogenetic classification of the Cordycipitaceae (Kepler et al. 2017; Kuephadungphan et al. 2019). The taxa of the new species were distributed in the *Gibellula* clade which was strongly supported (100%) and inferred as a monophyletic group. *Gibellula pigmentosinum* was found to be very close to Gibellula cf. alba by forming a strong supported clade together. *Gibellula fusiformispora* was inferred as the phylogenetic sister of *G. cebrennini*, whereas *G scorpioiodes* formed a distinct well-supported sister clade to these species.

**Table 1.** List of taxa included in the phylogenetic analysis and their GenBank accession numbers. The isolates representing four new species and other sequences generated in this study are marked in bold.

Species	Code	GenBank accession numbers					References
		ITS	LSU	*TEF1*	*RPB1*	*RPB2*	
*Akanthomyces aculeatus*	HUA 772	KC519371	KC519370	KC519366	–	–	Sanjuan et al. 2014
*A. aculeatus*	HUA 186145	–	MF416520	MF416465	–	–	Kepler et al. 2017
*A. sabanensis*	ANDES-F 1014	KC633245	KC633248	KC875221	–	–	Chirivi-Salomon et al. 2015
*A. sabanensis*	ANDES-F 1024^T^	KC633232	KC875225	KC633266	–	KC633249	Chirivi-Salomon et al. 2015
*Beauveria bassiana*	ARSEF 7518	HQ880762	–	HQ880975	HQ880834	HQ880906	Rehner et al. 2011
*B. bassiana*	ARSEF 1564^T^	NR111594	–	HQ880974	HQ880833	HQ880905	Rehner et al. 2011
*Cordyceps militaris*	OSC 93623	JN049825	AY184966	DQ522332	DQ522377	AY545732	Kepler et al. 2012; Spatafora et al. 2007; Sung and Spatafora 2004
*C. militaris*	ARSEF 5050	HQ880829	–	HQ881020	HQ880901	HQ880973	Rehner et al. 2011
*C. farinosa*	CBS 111113^T^	AY624181	MF416554	MF416499	MF416656	MF416450	Luangsa-ard et al. 2005; Kepler et al. 2017
*C. javanica*	CBS 134.22^T^	NR111172	NG059048	MF416504	MF416661	MF416455	Luangsa-ard et al. 2005; Kepler et al. 2017
***C. javanica***	BCC 26304	MH532851	**MH394660**	MH521903	**MH521825**	**MH521868**	Helaly et al. 2019, **this study**
*Blackwellomyces cardinalis*	OSC 93609^T^	–	AY184962	DQ522325	DQ522370	DQ522422	Sung and Spatafora 2004; Spatafora et al. 2007
*B. cardinalis*	OSC 93610	JN049843	AY184963	EF469059	EF469088	EF469106	Kepler et al. 2012; Sung and Spatafora 2004; Sung et al. 2007
*Engyodontium aranearum*	CBS 309.85	JN036556	AF339526	DQ522341	DQ522387	DQ522439	Spatafora et al. 2007; Sung et al. 2001
*E. aranearum*	CBS 658.80	LC092897	LC092916	–	–	–	Tsang et al. 2016
*Hevansia novoguineensis*	CBS 610.80^T^	MH532831	MH394646	MH521885	–	MH521844	Helaly et al. 2019; Mongkolsamrit et al. in press
*H. novoguineensis*	NHJ 4314	–	–	EU369012	EU369051	EU369071	Johnson et al. 2009
*H. novoguineensis*	NHJ 11923	–	EU369032	EU369013	EU369052	EU369072	Johnson et al. 2009
***H. novoguineensis***	BCC 47881	JX192685	**MH394650**	**MH521886**	**MH521807**	**MH521845**	Helaly et al. 2017, **this study**
*H. cinerea*	NHJ 3510	–	–	EU369009	EU369048	EU369070	Johnson et al. 2009
Gibellula cf. alba	NHJ 11679	–	–	EU369016	EU369054	–	Johnson et al. 2009
***G. cebrennini***	**BCC 32072**	**MT477067**	–	**MT503326**	–	–	**This study**
***G. cebrennini***	**BCC 39705**	**MH532874**	**MH394673**	**MH521895**	**MH521822**	**MH521859**	**This study**
***G. cebrennini***	**BCC 53551**	**MT477068**	–	**MT503327**	–	–	**This study**
***G. cebrennini***	**BCC 53605^T^**	**MT477069**	**MT477062**	**MT503328**	**MT503321**	**MT503336**	**This study**
G clavulifera var. alba	ARSEF 1915^T^	–	DQ518777	DQ522360	DQ522408	DQ522467	Spatafora et al. 2007
***G. fusiformispora***	**BCC 45076**	**MH532882**	–	–	**MH521823**	**MH521860**	**This study**
***G. fusiformispora***	**BCC 56802^T^**	**MT477070**	**MT477063**	**MT503329**	**MT503322**	**MT503337**	**This study**
*G. gamsii*	BCC 27968^T^	MH152529	MH152539	MH152560	MH152547	–	Kuephadungphan et al. 2019
*G. gamsii*	BCC 28797	MH152531	MH152541	MH152562	MH152549	MH152557	Kuephadungphan et al. 2019
*G leiopus*	BCC 16025	–	MF416548	MF416492	MF416649	–	Kepler et al. 2017
*G longispora*	NHJ 12014	–	–	EU369017	EU369055	EU369075	Johnson et al. 2009
*G pulchra*	NHJ 10808	–	EU369035	EU369018	EU369056	EU369076	Johnson et al. 2009
***G. pigmentosinum***	**BCC 38246**	MH532872	**MH394672**	MH521893	**MH521800**	**MH521855**	Helaly et al. 2019, **this study**
***G. pigmentosinum***	**BCC 39707**	MH532875	**MH394674**	MH521894	**MH521801**	**MH521856**	Helaly et al. 2019, **this study**
***G. pigmentosinum***	**BCC 41203^T^**	**MT477071**	–	**MT503330**	**MT503323**	–	**This study**
***G. pigmentosinum***	**BCC 41870**	**MT477072**	**MT477064**	**MT503331**	**MT503324**	–	**This study**
***G. scorpioides***	**BCC 13020**	**MT477073**	**MH394686**	**MH521901**	**MH521814**	–	**This study**
***G. scorpioides***	**BCC 43298**	**MT477074**	**MH394677**	**MH521900**	**MH521816**	**MH521858**	**This study**
***G. scorpioides***	**BCC 45127**	**MT477075**	–	**MT503332**	–	–	**This study**
***G. scorpioides***	**BCC 47514**	**MT477076**	–	**MT503333**	–	–	**This study**
***G. scorpioides***	**BCC 47530**	**MT477077**	**MT477065**	**MT503334**	–	**MT503338**	**This study**
***G. scorpioides***	**BCC 47976^T^**	**MT477078**	**MT477066**	**MT503335**	**MT503325**	**MT503339**	**This study**

### Taxonomy

#### Gibellula cebrennini

Taxon classificationFungiHypocrealesCordycipitaceae

Tasanathai, Kuephadungphan & Luangsa-ard sp. nov.

3AF9BF90-04AF-5B02-ABB8-BBCC020F3D2F

835113

Figure 2

##### Typification.

Thailand, Nakhon Ratchasima, Khao Yai National Park, Mo Sing To Nature Trail; 14°711'N, 101°421'E; on Cebrenninus cf. magnus (Thomisidae, Araneae) attached to the underside of unidentified dicot leaf; 20 June 2012; K. Tasanathai, S. Mongkolsamrit, A. Khonsanit, W. Noisripoom, P. Srikitikulchai, K. Sansatchanon, R. Somnuk (Holotype no. BBH 35749, ex-type culture no. BCC 53604, isolated from ascospores and BCC 53605, isolated from conidia). GenBank (BCC 53605): ITS = MT477069, LSU = MT477062, *TEF1* = MT503328, *RPB1* = MT503321, *RPB2* = MT503336.

##### Etymology.

Refers to its spider host.

##### Description.

**Figure 2.**
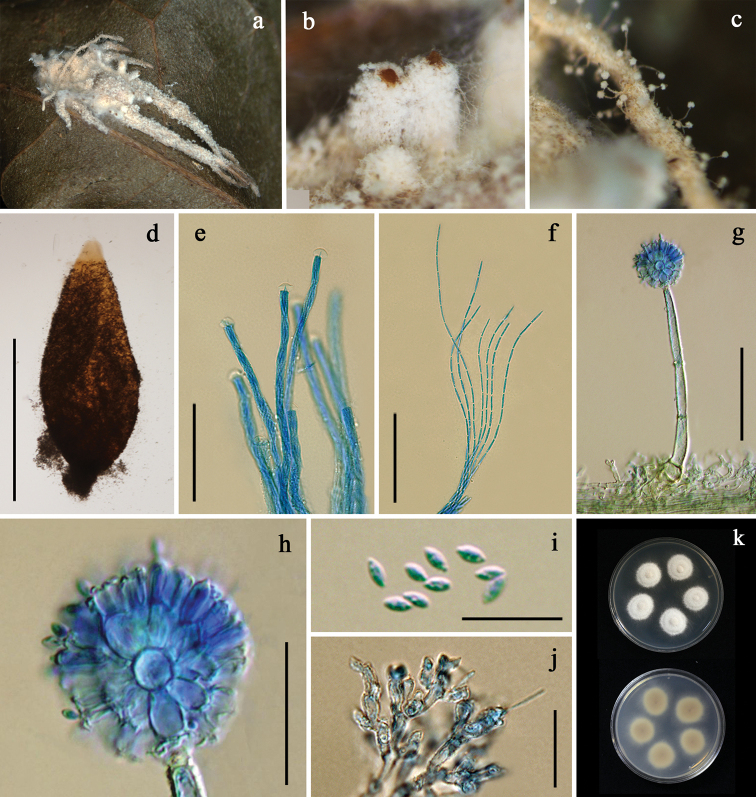
*Gibellula cebrennini***a** fungus on spider (BBH 35749) **b** perithecia **c** a part of synnema showing conidiophores **d** perithecium **e** asci with apical apparatus **f** ascospores **g** conidiophore **h** conidial head **i** conidia **j** granulomanus-like asexual morph **k** colonies obverse and reverse on PDA at 25 °C after 28 days. Scale bars: 1 mm (**d**); 50 μm (**e–f, g**); 20 μm (**h, j**); 10 μm (**i**).

*Synnema* arising from white to cream mycelial mat completely covering the spider host, cylindric, white to cream, slightly enlarged toward the sterile tip, consisting of multiseptate somewhat loosely bound longitudinal hyphae (Fig. 2a, c). *Conidiophores* scattered, arising from a network of hyphae loosely attached to the surface of the synnema, occasionally from a mycelium covering the host, (45–)95–139(–150) × (5–)5.5–7(–8) μm, verrucose, multiseptate, tapering abruptly to a short distinct neck, enlarging into a broadly ellipsoid to globose vesicle, (4.5–)5.5–7.5(–8.5) μm in diam (Fig. 2g). *Metulae* borne on vesicle, broadly obovoid to obovoid, (5–)6–7.5(–9) × (3–)4.5–6(–6.5) μm, bearing a group of narrowly obovoid phialides, thickened towards papillate apices, (4–)5.5–7.5(–9) × 1.5–2.5(–3.5) μm (Fig. 2h). *Vesicle*, metulae and phialides forming spherical heads, (23–)24–29.5(–33.5) μm in diam (Fig. 2h). *Conidia* fusiform, (4–)5.5–7.5(–9) × 1.5–2.5(–3.5) μm (Fig. 2i). *Perithecia* developed on subiculum of the host, arranged sparingly, occasionally crowded, superficial with a loose covering of cream mycelia, reddish yellow, ovoid, (1,150–)1,209–1,400(–1,411) × (375–)427–505(–575) μm (Fig, 2b, d). *Asci* over 600 μm long, (3.5–)4–5(–6) μm wide, ascus cap, (6–)7–8.5(–10) × (3.5–)4–4.5(–5) μm (Fig. 2e). *Ascospores* bacilliform, multiseptate, whole, over 570 μm long, 1–1.5 μm wide (Fig. 2f). Granulomanus-like asexual morph often occurring on the mycelial mat covering the host body. Polyblastic and irregularly shaped phialides developing multiple denticles, each bearing filiform conidium, (6–)7.5–10(–12) × 1–1.5 μm (Fig. 2j).

##### Culture characteristics.

Colonies derived from conidia, on PDA slow-growing, attaining a diam of 1.4±0.05 cm in 4 weeks at 25 °C, white, velvety; reverse cream, becoming light brown with age toward center (Fig. 2k). Sporulation not observed.

##### Additional specimen examined.

Thailand, Nakhon Ratchasima, Khao Yai National Park, Kong Kaeo Waterfall; 14°711'N, 101°421'E; on Araneida, underside of unidentified dicot leaf; 28 November 2006; K. Tasanathai, W. Chaygate, B. Thongnuch (BBH 18890, BCC 23863); Mo Sing To Nature Trail; 14°711'N, 101°421'E; on Cebrenninus cf. magnus, underside of unidentified dicot leaf; 18 June 2008; J. Luangsa-ard, K. Tasanathai, S. Mongkolsamrit, B. Thongnuch, P. Srikitikulchai, R. Ridkaew, W. Chaygate, R. Promharn (BBH 24673, BCC 32072). On Cebrenninus cf. magnus, underside of unidentified dicot leaf; 11 September 2009; K. Tasanathai, P. Srikitikulchai, S. Mongkolsamrit, T. Chohmee, R. Ridkaew (BBH 32685, BCC 39705 and BCC 39706). On Cebrenninus cf. magnus, underside of unidentified dicot leaf; 6 June 2012; K. Tasanathai, S. Mongkolsamrit, A. Khonsanit, W. Noisripoom, P. Srikitikulchai (BBH 32589, BCC 53551).

#### Gibellula fusiformispora

Taxon classificationFungiHypocrealesCordycipitaceae

Tasanathai, Kuephadungphan & Luangsa-ard sp. nov.

85B98007-DE5B-5741-AEF0-4585383FA25A

835114

Figure 3

##### Typification.

Thailand, Chiang Mai, Chiang Dao District, Ban Huathung; 19°420'N, 98°971'E; on Araneida attached to the underside of unidentified dicot leaf; 5 October 2012; K. Tasanathai, A. Khonsanit, W. Noisripoom, P. Srikitikulchai, R. Promharn (Holotype no. BBH 32918, ex-type culture no. BCC 56802, isolated from conidia) GenBank: ITS = MT477070, LSU = MT477063, *TEF1* = MT503329, *RPB1* = MT503322, *RPB2* = MT503337.

##### Etymology.

Refers to the fusiform part-spores.

##### Description.

**Figure 3.**
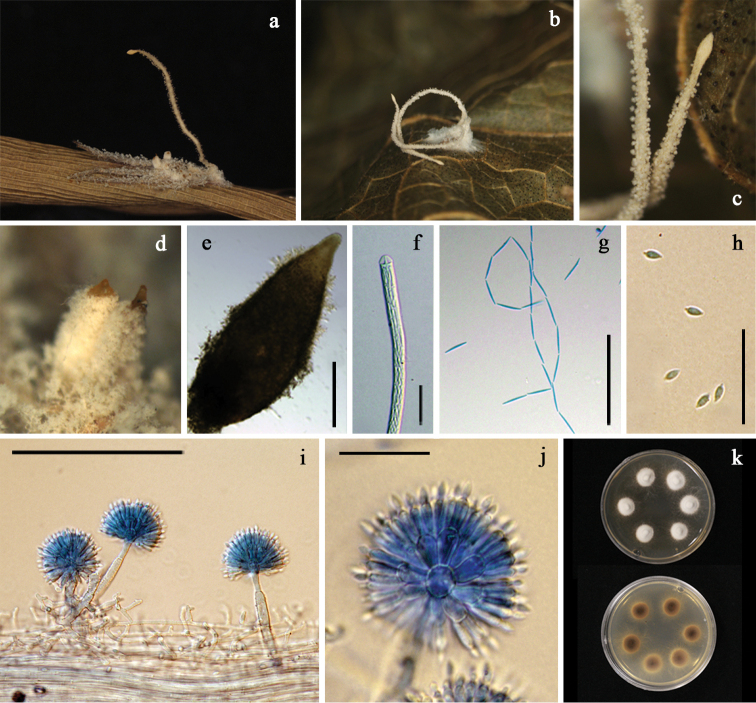
*Gibellula fusiformispora***a** fungus on a spider (BBH 38838) **b** fungus on a spider (BBH 32918) **c** synnemata (BBH 32918) **d, e** perithecia (BBH 38838) **f** ascus (BBH 38838) **g** ascospores (BBH 38838) **h** conidia (BBH 32918) **i** conidiophores showing conidial heads (BBH 32918) **j** conidial head bearing conidia (BCC 32918) **k** colonies obverse and reverse on PDA at 25 °C after 20 days. Scale bars: 250 μm (**e**); 100 μm (**i**); 50 μm (**g**); 20 μm (**f, h, j**).

Spiders totally covered by the white to cream mycelial mat. A single synnema or synnemata in pairs cream to light brown, often darker than the mycelia covering the host, narrowing toward the apex and terminating in a swollen sterile tip with acute apex (Fig. 3a–c). *Conidiophores* arising laterally from the outer layer of synnemata, crowded, (23–)31–53(–83) × (4–)5.5–6.5(–7.5) μm, mostly verrucose, occasionally slightly roughed for very short conidiophores, abruptly narrowing to a slender apex and forming a globose to subglobose vesicle (Fig. 3c, i). *Vesicles* 6–7(–8) μm in diam, each bearing a number of metulae (Fig. 3j). *Metulae* obovoid to broadly obovoid, (7–)7.5–9(–10) × (4.5–)5–5.5(–6) μm (Fig. 3j). *Phialides* borne on metulae, narrowly obovoid, 7–8.5(–10) × 2–3 μm bearing fusiform to broadly fusiform conidia, (3.5–)4–5(–6) × 1.5–2(–2.5) μm (Fig. 3h, j). *Conidial heads* spherical, (31–)32–34.5(–37) μm in diam (Fig. 3j). *Perithecia* mostly appearing in pairs, ovoid, superficial with a loose covering of white to cream mycelia, reddish yellow, up to 1,000 μm in length, 320–350 μm in width (Fig. 3d–e). *Asci* 600–700 × 7–8 μm (Fig. 3f). *Ascospores* often disarticulating into part-spores. *Part-spores* fusiform, 12–15 × 2–3 μm (Fig. 3g). Granulomanus-like asexual morph absent.

##### Culture characteristics.

Colonies derived from conidia, on PDA slow-growing, attaining a diam of 1.1±0.03 cm in 20 days at 25 °C, white, velvety; reverse cream, becoming light brown with age toward center (Fig. 3k). Sporulation not observed.

##### Additional specimen examined.

Thailand, Chiang Mai, Chiang Dao District, Ban Huathung; 19°420'N, 98°971'E; on Deinopidae (Araneae) attached to the underside of unidentified monocot leaf; 23 September 2010; K. Tasanathai, P. Srikitikulchai, A. Khonsanit, K. Sansatchanon (BBH 38838, BCC 45076 and BCC 45077).

##### Notes.

The sexual morph of *G. fusiformispora* is extremely close to *Torrubiella ellipsoidea* (Kobayasi and Shimizu 1982) in producing slightly curved fusiform part-spores of maximum 3 µm wide, whereas those of *G. fusiformispora* are almost two times wider. Considering the *Gibellula* conidial state, *G. fusiformispora* resembled *G. cebrennini* by forming synnema with sterile swollen tip, aspergillate conidiophores and fusiform conidia. However, *G. fusiformispora* can be easily recognized by having much shorter conidiophores, and the production of more than one synnema on the spider hosts.

#### Gibellula pigmentosinum

Taxon classificationFungiHypocrealesCordycipitaceae

Tasanathai, Kuephadungphan & Luangsa-ard sp. nov.

5335E10A-EAD1-526F-8D8B-10719F05BBE2

835112

Figure 4

##### Typification.

Thailand. Nakhon Ratchasima, Khao Yai National Park, Mo Sing To Nature Trail; 14°711'N, 101°421'E; on *Storenomorpha* sp., attached to underside of unidentified dicot leaf; 10 February 2010; K. Tasanathai, P. Srikitikulchai, S. Mongkolsamrit, T. Chohmee, R. Ridkaew, A. Khonsanit (Holotype no. BBH 28509, ex-type culture no. BCC 41203, isolated from ascospores and BCC 41204, isolated from conidia). GenBank (BCC 41203): ITS = MT477071, *TEF1* = MT503330, *RPB1* = MT503323.

##### Etymology.

Refers to the capability of the fungus to produce pigmentosins.

##### Description.

**Figure 4.**
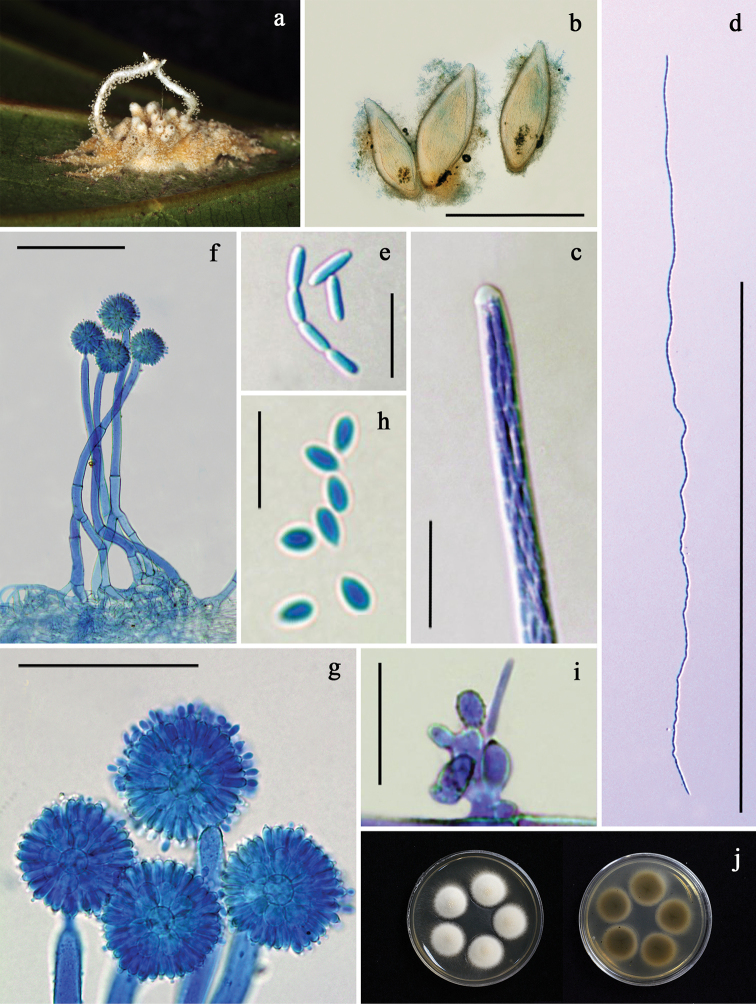
*Gibellula pigmentosinum***a** fungus on spider (BBH 28509); **b** perithecia; **c** an ascus with an apical apparatus; **d** ascospore; **e** part-spores; **f** conidiophores; **g** conidial heads; **h** conidia; **i** granulomanus-like asexual morph; **j** colonies obverse and reverse on PDA at 25 °C after 28 days. Scale bars: 1 mm (**b**); 500 μm (**d**); 100 μm (**f**); 50 μm (**g**); 20 μm (**c, i**); 10 μm (**e, h**).

Spider host completely covered by white to yellowish-white mycelial mat. *Synnemata* solitary or in pairs, cylindrical, white, becoming yellowish-white at the base (Fig. 4a). *Conidiophores* arising along the entire length of the outer hyphae of synnemata and from the mycelia covering the host, crowded, smooth to verrucose, (55–)97.5–170(–226) × (5–)7–10(–12.5) µm, narrowing to a slender apex, and terminating in a swollen vesicle, metulae, phialides bearing conidia, forming a spherical conidial head (Fig. 4a, f). *Conidial heads* (25–)30–39(–45) µm diam (Fig. 4g). *Vesicles* mostly globose, (4.5–)5.5–9(–10) µm diam (Fig. 4g). *Metulae* borne on a vesicle, broadly obovoid, (5.5–)6–8(–10) × (3–)4–6(–7.5) µm (Fig. 4g), bearing phialides. *Phialides* obovoid to clavate, with a distinct short neck, (5–)5.5–8(–9) × 2–3(–4.5) µm (Fig. 4g). *Conidia* produced on a phialide, obovoid with an acute apex, (2.5–)3.5–5(–5.5) × 1–2(–3) µm (Fig. 4h). *Perithecia* produced on the mycelial mat on the head and body of the spider, scattered, superficial with loose mycelia covering only the bottom one-fourth of the perithecium, ovoid, reddish-yellow, (790–)882–1,117(–1,150) × 300–443(–475) μm (Fig. 4a, b). *Asci* cylindrical, 700–750 μm long, (4.5–)5–6(–7) μm wide, ascus cap (4–)5.5–6.5(–7) × 3.5–4(–5.5) μm (Fig. 4c). *Ascospores* filiform, multiseptate, arranged in parallel rows, (666–)670–727(–730) × 2–3 μm, often breaking into 128 part-spores (Fig. 4d). *Part-spores* bacilliform with apices rounded, (3.5–)4–7(–9) × 1–1.5(–3) μm (Fig. 4e). Granulomanus-like asexual morph occasionally present, forming irregularly branched hyphae bearing mono- or polyblastic phialides. *Phialides* irregularly in shape, mostly smooth, with one or more conspicuous denticles. A *conidium* borne on each denticle, long, filiform, (16–)16.5–21.5(–22.5) × 1–1.5 μm (Fig. 4i).

##### Culture characteristics.

Colonies derived from ascospores, on PDA slow-growing, attaining a diam of 1.5±0.2 cm in 4 weeks at 25 °C, white, floccose; reverse light brown, darkening with age toward center (Fig. 4j). Sporulation not observed.

**Additional specimens examined.** Thailand, Nakhon Ratchasima, Khao Yai National Park, Mo Sing To Nature Trail; 14°711'N, 101°421'E; on *Storenomorpha* sp., underside of unidentified dicot leaf; 13 August 2009; K. Tasanathai, P. Srikitikulchai, S. Mongkolsamrit, T. Chohmee, R. Ridkaew (BBH 26516, BCC 38246 and BCC 38955); on Araneida, underside of unidentified dicot leaf; 11 September 2009; K. Tasanathai, P. Srikitikulchai, S. Mongkolsamrit, T. Chohmee, R. Ridkaew (BBH 27081, BCC 39707 and BCC 39708); on *Storenomorpha* sp., underside of unidentified dicot leaf; 7 April 2010; K. Tasanathai, S. Mongkolsamrit, T. Chohmee, A. Khonsanit, R. Ridkaew (BBH 28533, BCC 41870 and BCC 41871).

##### Notes.

*Gibellula pigmentosinum* shares similarity with *G pulchra* (Mains 1950) in producing cylindric, yellowish white synnemata bearing aspergillate conidiophores with fusoid-ellipsoid conidia and superficial, reddish brown, ovoid perithecia containing bacilliform part-ascospores. The synnemata in *G pulchra* are more copious and sometimes more violaceous than in *G. pigmentosinum*. Remarkably, *G. pigmentosinum* distinctly differs from *G pulchra* in having a granulomanus-like conidial state.

#### Gibellula scorpioides

Taxon classificationFungiHypocrealesCordycipitaceae

Tasanathai, Khonsanit, Kuephadungphan & Luangsa-ard sp. nov.

9B6A9DAA-741F-5609-B789-ADAE8F27A665

835115

Figure 5

##### Typification.

Thailand, Nakhon Ratchasima, Khao Yai National Park, Mo Sing To Nature Trail, 14°711'N, 101°421'E; on *Portia* sp. attached to the underside of unidentified dicot leaf; 1 June 2011; K. Tasanathai, P. Srikitikulchai, S. Mongkolsamrit, A. Khonsanit, K. Sansatchanon, W. Noisripoom (Holotype no. BBH 31439, ex-type culture no. BCC 47975, isolated from ascospores and BCC 47976, isolated from conidia) GenBank (BCC 47976): ITS = MT477078, LSU = MT477066, *TEF1* = MT503335, *RPB1* = MT503325, *RPB2* = MT503339.

##### Etymology.

Refers to the outer appearance of the fungus resembling the posture of a scorpion.

##### Description.

**Figure 5.**
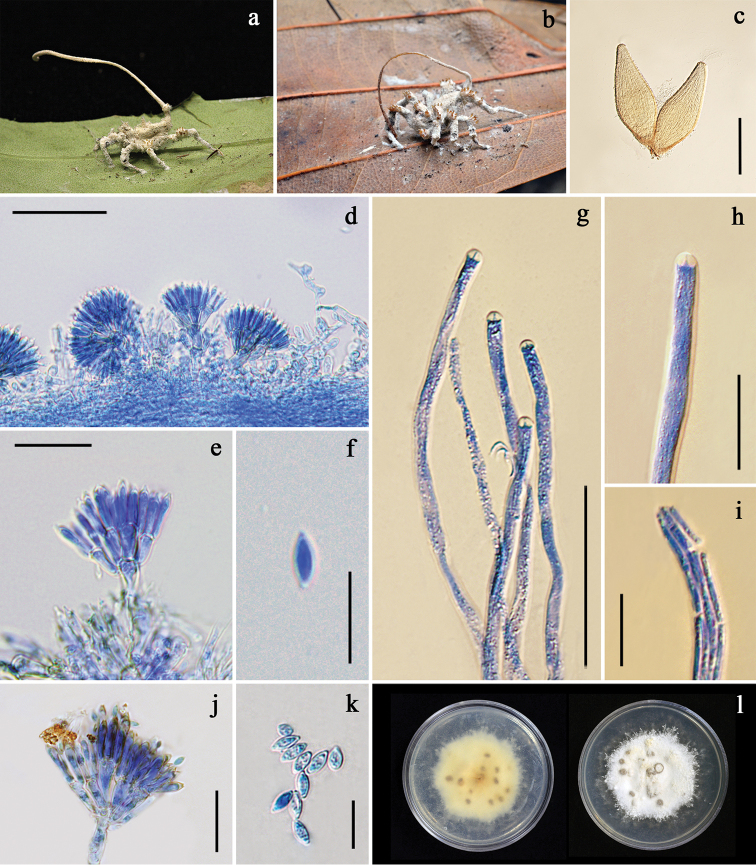
*Gibellula scorpioides***a** fungus on a spider (BBH 29669) **b** fungus on a spider (BBH 31439) **c** perithecia (BBH 31439) **d** conidiophores arising on synnema (BBH 29669) **e** penicillate conidiophore (BBH 29669) **f** conidia (BBH 29669) **g** asci (BBH 31439) **h** ascus with apical apparatus (BBH 31439) **i** ascospores (BBH 31439) **j** penicillate conidiophore produced on PDA**k** conidia on PDA**l** colonies obverse and reverse on PDA at 25 °C after 4 months. Scale bars: 500 μm (**c**); 50 μm (**d, g**); 20 μm (**e, h–j**); 10 μm (**f, k**).

White to grayish- or brownish-white mycelial mat velvety, completely covering the spider host, firmly attaching the underside of living leaf by the mycelia covering its legs (Fig. 5a, b). *Synnema* solitary, arising from the posterior of the host abdomen, cylindrical, consisting of a compact bundle of parallel hyphae, 15–20 mm long with blunt tip. *Conidiophores* arising laterally from synnema, stout, smooth, mostly biverticillate, 20–29(–30) × 4 μm (Fig. 5d). *Vesicles* absent or hardly developed, bearing multiple metulae. *Metulae* obovoid, slightly broadening toward the base, (7–)9.5–12.5(–15) × (2–)3–5(–7) μm (Fig. 5e). A number of phialides borne on each metula, broadly cylindrical, abruptly tapering toward the apex, forming thickened distinct short neck, (9–)10–12.5(–14) × (2–)2.5–3.5(–4) μm, each bearing a conidium (Fig. 5e). *Conidia* fusiform, 5–7(–9) × (1.5–)2–3 μm (Fig. 5f). Sexual morph occasionally present. *Perithecia* occurring on the mycelial mat covering the host legs, occasionally on synnema particularly at base, superficial, mostly arranged in groups, ovoid, reddish yellow or light honey-brown, one-third immersed in the loose network of mycelia, 750–836(–870) × 310–361(–380) μm (Fig. 5c). *Asci* over 550 μm in length, (3–)4–5.5(–7) μm in width, ascus tip (4–)4.5–5 × 3–3.5(–4) μm (Fig. 5g, h). *Ascospores* often breaking into part-spores. *Part-spores* bacilliform, (9–)10–15(–22) × 1.5–2 μm (Fig. 5i). Granulomanus-like asexual morph absent.

##### Culture characteristics.

Colonies derived from conidia, on PDA slow-growing, attaining a diam of 1.5±0.1 cm in 4 weeks at 25 °C, floccose, forming irregular margin, white, reverse cream, darkening toward center with age (Fig. 5l). Sporulation occurring after 3–4 months with the absence of synnema, forming a group of conidiophores, grey and scatter. *Conidiophores* biverticillate. *Vesicles* absent or hardly developed. *Metulae* obovoid, (10–)11–14.5(–16) × 3–5.5(–7) μm, each bearing cylindrical *phialides*, (10–)11.5–14(–16) × 3–4 μm. *Conidia* fusiform, 5–6(–7) × 3–3.5(–4) μm.

**Additional specimens examined.** Thailand, Chumphon, Phato District, Phato Watershed Conservation and Management Unit; 9°784'N, 98°699'E; on *Portia* sp., underside of unidentified dicot leaf; 10 March 2011; K. Tasanathai, P. Srikitikulchai, A. Khonsanit, K. Sansatchanon, D. Thanakitpipattana (BBH 30499, BCC 47530). Nakhon Ratchasima, Khao Yai National Park, Mo Sing To Nature Trail; 14°711'N, 101°421'E; on *Portia* sp., underside of unidentified dicot leaf; 1 June 2011; K. Tasanathai, P. Srikitikulchai, S. Mongkolsamrit, A. Khonsanit, K. Sansatchanon, W. Noisripoom (BBH 29669, BCC 43298).

##### Notes.

The morphology of *G. scorpioides* appeared to be very close to G clavulifera var. clavulifera (Samson and Evans 1977), G clavulifera var. major (Tzean et al. 1997) and G clavulifera var. alba (Humber and Rombach 1987). The penicillate conidiophores were largely absent from the whip-like stroma in G clavulifera var. alba but distinctly present on a synnema of *G. scorpioides*. Based on a comparison of microscopic characteristics among *G. scorpioides*, varieties *clavulifera*, *major* and *alba*, the latter three were found to produce much longer conidiophores (up to 100 μm) than *G. scorpioides* (20–29(–30) × 4 μm) while the other characters such as metulae, phialides as well as conidia were considered to be not significantly different in both shape and size. Considering the presence of the torrubiella-like sexual morph, perithecia of G clavulifera var. alba were produced sparingly and separately on the host abdomen while those of *G. scorpioides* distinctly appeared in groups, only on the spider’s legs and basally on synnema. Nevertheless, an examination of additional specimens has led us to conclude that the sexual morph is not always present in *G. scorpioides*.

## Discussion

A torrubiella-like sexual morph is well-known to be connected with *Gibellula* (Evans 2013; Kepler et al. 2017; Shrestha et al. 2019). Shrestha and colleagues (2019) recently reviewed spider-pathogenic fungi within Hypocreales including *Gibellula* where its sexual morph links are listed. *Torrubiella globosa* Kobayasi & Shimizu, *Torrubiella globosostipitata* Kobayasi & Shimizu, Torrubiella arachnophila var. pulchra Mains and *Torrubiella gibellulae* Petch were synonymized with *G pulchra*, species where their conidial and torrubiella-like sexual morphs often concurrently occur on the same substrates. *Gibellula pigmentosinum* appeared to be remarkably close to *G pulchra* in producing nearly identical microscopic characters in both shapes and sizes. Nonetheless, *G. pigmentosinum* distinctly differs from *G pulchra* in having a granulomanus-like conidial state. Considering its phylogenetic placement, *G. pigmentosinum* was significantly placed far from the taxon representing *G pulchra* which supported the morphological differences between them. Noticeably, *G. pigmentosinum* formed a very strongly supported clade together with Gibellula cf. alba. It is interesting that Gibellula cf. alba was not proposed as a species and it is unfortunate that the herbarium specimen of NHJ 11679 is no longer in a good condition. According to its placement in the RAxML/Bayesian tree inferred from multiple loci (Fig. 1), Gibellula cf. alba NHJ 11679 could unambiguously be assigned to *G. pigmentosinum*.

The morphological resemblance between *G. cebrennini* and *G. fusiformispora* as well as a multi-gene phylogenetic analysis indicate a very close relationship among these species. Moreover, they can be distinguished from each other by the length of conidiophores, the shape of ascospores as well as the presence of a granulomanus-like conidial state.

In nature, a torrubiella-like sexual morph may occur on spider hosts without the presence of *Gibellula*. It may be premature to assign the new species to *Gibellula* on the basis of sexual morph, when more than one asexually reproductive genus are known to be linked to a torrubiella-like sexual morph. *Gibellula cebrennini* and *Akanthomyces thailandicus* (Mongkolsamrit et al. 2018) are good examples of such a phenomenon. Based on an investigation of Thai specimens, *G. cebrennini* tended to produce torrubiella-like perithecia on the spider hosts in the absence of *Gibellula* and granulomanus-like asexual morphs, whereas *A. thailandicus* is an obligate parasite of spiders, of which only its torrubiella-like sexual morph has so far been recorded. These characteristics could lead to misidentification between these two species. The size and shape of part-spores are considered as the only morphological characters that have potential in species discrimination according to the evidence that *G. cebrennini* mostly produces longer bacilliform part-spores than *A. thailandicus*. In *G. cebrennini* and *G. fusiformispora*, it may also be difficult to discriminate them at first glance as the only distinguishing feature is the shape of their part-spores. These similiarities, and the occasional overlap in shape and size of morphological characters, were also demonstrated by Khonsanit et al. (2019) in the *Ophiocordyceps irangiensis* and *O. myrmecophila* species complex, by Mongkolsamrit et al. (2019) in *Paraisaria phuwiangensis* and *P. yodhathaii*, by Luangsa-ard et al. (2018) in *Ophiocordyceps* spp. with superficial perithecia, and Tasanathai et al. (2019) among termite pathogens in *Ophiocordyceps*. To improve species delimitation among closely related species with such very low morphological differentiation, integrative taxonomy combining a variety of data such as molecular, chemical, biogeographical, ecological characters, etc. is suggested to be very useful (Pante et al. 2015).

It has been over a half century since host specialization was suggested as one of the taxonomic criteria for parasitic fungi (Johnson 1968). In most cases of fungi that have a narrow host range or are restricted to a single host species, host specificity is considered as an important feature that can be used for identification at the species level (Vialle et al. 2013). In the case of invertebrate-pathogenic fungi, host specificity is usually taken into account mostly to evaluate their virulence and potential in terms of using them as biocontrol agents. For taxonomic purposes, Kobmoo and co-workers (2012) proved that host specificity has great potential for reflecting the divergent evolution of the ant-parasitic *Ophiocordyceps unilateralis*. Their success has therefore driven us to put effort for the first time to define the host species of *Gibellula*.

Bishop (1990), Hughes et al. (2016), and Savić et al. (2016) reported spider hosts of *Gibellula* distributed among 11 families consisting of Anyphaenidae, Agelinidae, Araneidae, Corinnidae, Linyphiidae, Pholcidae, Salticidae, Sparassidae, Theridiidae, Thomisidae, Zodariidae which represent approximately 10% of described families worldwide (World Spider Catalog 2020). Herein, three of those including Salticidae, Thomisidae and Zodariidae were found to be the hosts of *G. scorpioides*, *G. cebrennini* and *G. pigmentosinum*, respectively, whereas the family Deinopidae is reported here for the first time as a *Gibellula* host for *G. fusiformispora*.

According to the effort of putting toward species identification of spider hosts (Bristowe1930; Tikader 1965; Jocqué and Bosmans 1989; Deeleman-Reinhold 2001, 2009; Jocqué and Dippenaar-Schoeman 2007; Lehtinen et al. 2008; Benjamin 2011, 2016; Miller et al. 2012; Dhali et al. 2017; Jocqué et al. 2019), *G. pigmentosinum*, *G. cebrennini* as well as *G. scorpioides* were found to be exclusively specific to spider species with the exception of *G. fusiformispora* wherein only one specimen allowed us to identify the spider only to the family rank. Despite *Gibellula* being a well-known spider specialist, only Nentwig (1985), Hughes et al. (2016), and Savić et al. (2016) have ever indicated its hosts at genus or species ranks. In the current study, only the host of *G. cebrennini* could be identified to the species level as Cebrenninus cf. magnus, whereas hosts of *G. pigmentosinum* and *G. scorpioides* were assigned to *Storenomorpha* sp. (Zodariidae) and *Portia* sp. (Salticidae), respectively. One common problem is that the fungus tends to cover the host body completely, which can obscure the spider’s morphological features making identification infeasible. Tarsal claws and scopulae are important morphological features used to identify spider species (Wolff and Gorb 2012; Wolff et al. 2013; Labarque et al. 2017), and the morphology of spider feet was herein targeted and carefully studied. Since their legs appeared to be the only part that were slightly covered by fungal mycelia, it was thus considered to be the most significant character for distinguishing spider species infected by *Gibellula*. Host identification can be especially challenging for old fungal herbarium specimens that are dry and damaged. We suggest delivering specimens to arachnological taxonomists immediately after field work to allow the identification of spider hosts to species rank. Furthermore, as new species of spider continue to be described (e.g. Miller et al. 2012; Dhali et al. 2017; Jocqué et al. 2019), accurate taxonomy of spider hosts could be important for taxonomy of fungal pathogens on them.

It is notable that all seven host individuals used for identification in this study were found under a leaf. *Portia* sp. has a unique life history. These spiders are not only web invaders or cursorial hunters but are also web builders. Thus, they exist both on and off their own webs. Their webs are used for various activities including trapping, baiting, resting, molting, mating, oviposition, brooding (Jackson and Blest 1982; Jackson and Hallas 1986; Nelson and Jackson 2011a). They are day-active hunters and stay on their own webs at night (Barth 2002; Herland and Jackson 2004; Nelson and Jackson 2011b). Moreover, moribund web-building spiders infected by pathogenic fungi are presumed to stay motionless on their webs (Anotaux et al. 2016). This behaviour may promote growth and reproduction of *G. scorpioides* since spider silks possibly have antifungal properties (Tahir et al. 2015; Phartale et al. 2019). Another distinct feature of the spiders infected by *G. scorpioides* is its firm attachment to the substrate by only the mycelia growing over the tips of their legs allowing the host to sprawl and elevate their bodies upward. Interestingly, the host of *G. scorpioides*, *Portia* sp. is an araneophagic jumping spider that usually assumes such a posture during hunting (Forster and Murphy 1986). Furthermore, spiders are generally found dead with a posture of their legs flexed beneath their body (Pollard et al. 1987; Pizzi 2006; Starr and Taggart 2006). Such dead posture may also support growth and reproduction of *G. scorpioides* by keeping host from predation (Deeleman-Reinhold 2001; Honma et al. 2006), at least at the beginning stage of a fungal growth. From these observations, we believe that the fungus may influence the behaviour of the spider host by forcing it to stay firmly in place and assume an active posture during infection.

Since *G. scorpioides* can be cultured, it is possible to apply fungal spores to *Portia* spiders and study the spider-fungus interaction. It will be particularly interesting to investigate death sites, on or off web and death posture, resting or hunting postures between uninfected control and fungal infected spiders, which may give insight into behavioural manipulation before death by the fungus. Additionally, cultured *G. scorpioides* could be used to test the antifungal properties of *Portia* spider silk.

It is remarkable that our finding has revealed the high possibility to incorporate the host specificity in molecular and morphological criteria for classification and identification of *Gibellula*.

The biggest challenge for molecular phylogeny-based classification of *Gibellula* is the lack of reliable sequence data from type specimens. From sequences available in public databases, identities often appear erroneous, e.g. *G. clavispora* (Chen et al. 2016), *G. curvispora* (Han et al. 2013) and *G shennongjiaensis* (Zou et al. 2016) appeared to be closer to other ascomycetes than *Gibellula* based on ITS sequence data. In the past, no attempts were made to establish the described species as pure cultures, or the attempts failed, thus making molecular analysis impractical. The lack of sequence data from type strains of *Gibellula* makes it difficult to establish whether query sequences from new specimens represent new or rediscovered taxa.

Despite molecular phylogeny currently being the most powerful approach available in modern fungal classification and taxonomy (Ariyawansa et al. 2014), many attempts to incorporate alternative or polyphasic approaches, such as chemotaxonomy, have been made. This approach provides high-informative data to support morphological and molecular data for identifying fungal species, facilitates solving taxonomic problem as well as unraveling asexual morph-sexual morph links (Frisvad et al. 1990; Stadler et al. 2003; Helaly et al. 2018). As part of our ongoing research on taxonomy and secondary metabolite production of Thai invertebrate-pathogenic fungi, chemotaxonomy has been employed, resulting in the discovery of unprecedented secondary metabolites, including pigmentosin B from *G. pigmentosinum* and gibellamines from *G. gamsii* (Helaly et al. 2019; Kuephadungphan et al. 2019). These compounds were found to be species-specific and could be designated as chemotaxonomic markers for the species. However, it is premature to use such compounds as markers for *Gibellula* since the exploration of their secondary metabolite production is limited to only a few species. Chemotaxonomy must therefore be expanded to other taxa, in particular *G. cebrennini*, *G. fusiformispora* as well as *G. scorpioides*.

## Supplementary Material

XML Treatment for Gibellula cebrennini

XML Treatment for Gibellula fusiformispora

XML Treatment for Gibellula pigmentosinum

XML Treatment for Gibellula scorpioides
